# Green synthesis of TiO_2_ nanoparticles prepared from *Phyllanthus niruri* leaf extract for dye adsorption and their isotherm and kinetic studies

**DOI:** 10.1049/nbt2.12033

**Published:** 2021-03-22

**Authors:** Anitha Panneerselvam, Jeevanantham Velayutham, Sudha Ramasamy

**Affiliations:** ^1^ Department of Chemistry Government College of Engineering Salem Tamilnadu India; ^2^ Department of Chemistry Vivekanandha College of Arts and Sciences for Women Namakkal Tamilnadu India; ^3^ Department of Chemistry Gnanamani College of Technology Namakkal Tamilnadu India

## Abstract

Herein, the green synthesis of TiO_2_ nanoparticles using *Phyllanthus niruri* leaf extract was accomplished by the sol‐gel method. The structure and particle size of the synthesised TiO_2_ nanoparticles were characterised by X‐ray diffraction (XRD) analysis and the size was found to be 20 nm. The Fourier‐transform infrared spectra determined the existence of carboxyl and hydroxyl functional groups. The images from SEM analysis recommended a porous and heterogeneous surface. The methyl orange (MO) dye removal was examined using different parameters such as pH, time, dose, temperature and dye concentration. Maximum dye elimination percentage was achieved at pH 6.0 and 0.02 g as the optimum adsorbent dose. The kinetic analysis suggested that the pseudo‐second‐order kinetic model finely defines adsorption dynamics. Langmuir adsorption isotherm studies revealed endothermic monolayer adsorption of the methyl Orange dye. The negative value of ∆*G*° and positive value of ∆*H*° showed the spontaneous and endothermic adsorption method.

## 1 INTRODUCTION

In the present scenario, water pollution is a severe threat to the eco‐system, so it has become essential to remove the contaminants from the effluents before it is discharged into the natural water resources. Directly or indirectly, fresh water is polluted by the untreated industrial effluents from various production units and industries. Aquatic bodies and aquatic organisms are affected harmfully by one of the major contaminants that is organic aromatic dye discharged from the industries. Dyes possess a complex aromatic structure, which are difficult to be degraded and also have carcinogenic and mutagenic effects on human beings [1, 2, 3]. Anionic dyes are lethal, as they not only pollute the environment but also affect the entire food chain resulting in biomagnification. Methyl Orange (MO) is an anionic dye, which is used as a colouring agent for dying various materials and it is necessary to remove methyl orange from the industrial discharge before it flows into the sewage thereby ensuring environmental safety.

Nanotechnology is an emerging field of science and it creates impact in solving many issues related to health and energy towards the need of the society. Currently, the biosynthesis of nanoparticles has been considered as environmentally sound and safer and more cost‐effective alternative for chemical and physical production methods [4]. Plant extracts are mainly promising for ‘green’ production since they are freely available, cheap, and offer simplicity of use and scalability. Various methods including chemical and physical means, chemical reduction, sol–gel, solvothermal, hydrothermal and electrochemical reduction techniques are widely employed for the synthesis of nanomaterials. But, the above methods are costly, requires high energy, difficult to separate and potentially hazardous.

Numerous conventional techniques including coagulation [5, 6, 7], electrochemical destruction [8], ozonation [9], and ion exchange [10, 11] are examined to treat the dye contaminated waste water. Of all these, adsorption is considered as an effective technique because of its simple design, high‐level efficiency and its convenience in operating. Among physical, chemical and biological conventional methods of dye removal, physical adsorption is an effective method for fast removal of dyes from the effluents [12, 13]. Many adsorbents such as activated carbon [14], peat [15], silica [16], clay [17], strychnospotatorum seeds [18], orange peel [19], tamarind seeds [20], and waste materials [21] are available.

Due to growing responsiveness towards green chemistry, the biological process has led to the development of an environment‐friendly process for the synthesis of non‐toxic nanoparticles. A variety of biological resources are available in nature including plant product such as leaves, bark, roots, stems, peels, etc., could be employed for the green synthesis of non‐toxic nanoparticles. Green synthesis is one of the best methods and does not require any high cost equipment and hazardous chemicals [22, 23].

TiO_2_ NPs exhibit distinctive surface properties and morphologies and used in the preparation of fibres, powder, paint, food‐stuffs and also employed in the deprivation of poisonous chemicals in H_2_O. Many methods need high temperature, pressure and poisonous chemicals which bounds their manufacture and important medicinal usages. Therefore, an ecological and economical method is required in preparing these nanomaterials with slighter risks. This could be possibly accomplished by the green synthesis method. where uses such as illnesses, handling, production of medical apparatus, construction of energy and cultivation are carried out.

The aim of the present research is to prepare the green synthesis of TiO_2_ nanoparticles from the *Phyllanthus niruri* leaf extract for the reason that green preparation is advantageous, simple, operative and quick in bulk without considerably capital costs and synthesised TiO_2_ nanoparticles used as an adsorbent for the removal of methyl orange from aqueous solution. The adsorption kinetics, equilibrium and thermodynamics of MO onto TiO_2_ nanoparticles are also investigated.

## 2 MATERIALS AND METHODS

### 2.1 Reagents and Instruments

Methyl orange dye used in this study was purchased from Merck (Germany). All the reagents used in the experiments were of analytical grade (AR) and used without further purification. To prepare the stock solution, 0.1 g of methyl orange dye was dissolved in 1 L of deionised water. For working solutions, the intermediate solutions were diluted sequentially and prepared fresh as and when required. The solution's pH was adjusted with 0.1 N sodium hydroxide and 0.1 N hydrochloride solution and a pH metre was used to measure its concentration. The known concentrations of standard methyl orange dye solution were used to obtain a calibration curve before the measurement. Also, double beam UV‐Vis spectrometer (Shimadzu, Model UV 160, and Japan) at 464 nm was used to measure the residual dye concentration.

### 2.2 Green synthesis of TiO_2_ nanoparticles using *Phyllanthus niruri* leaf extract

**FIGURE 1:**
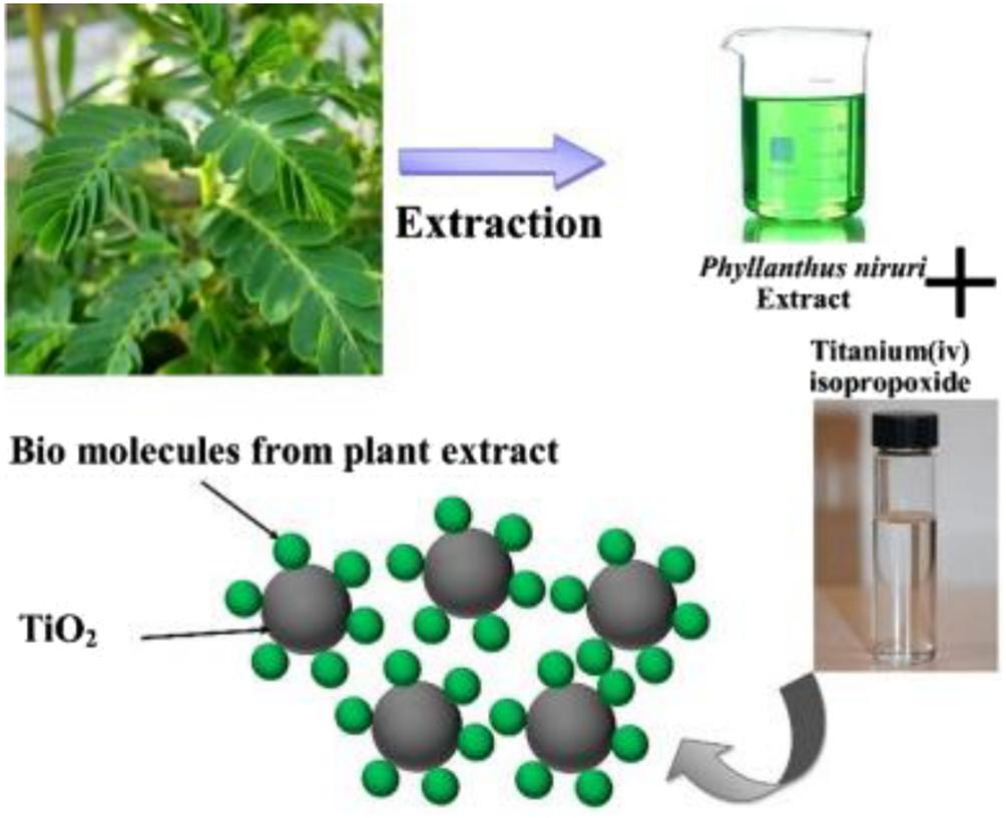
Green synthesis of TiO_2_ nanoparticles

Sol‐gel method [24] was utilised for the preparation of TiO_2_ nanoparticles using *Phyllanthus niruri* as shown in Figure 1. About 20 g of dried *Phyllanthus niruri* leaf powder was dissolved in 150 mL of distilled water and refluxed for 2 h using Soxhlet apparatus at 50°C. Then, the extract was filtered with Whatman filter paper and 20 mL of 1 mM titanium isopropoxide (TTIP) was added drop wise in 50 ml of the leaf extract followed by continuous stirring for 4 h at room temperature. After 4 h, the colour of the extract with TiO_2_ nanoparticles was changed to green. The solution was centrifuged at 5000 rpm for about 20 min to get sediment. At 80°C, the resultant product was dehydrated in the vacuum oven for 24 h that yielded TiO_2_ nanopowder. The TiO_2_ NPs were calcined at 600°C for 3 h. The calcined titanium dioxide nanopowder was used for further experiment.

### 2.3 Adsorption experimental analytical procedure

The effects of influential variables were investigated on the adsorption process, including the contact time (20–60 min), pH of solution (4–12), amount of adsorbent (0.01–0.04 g), dye concentrations (10–40 mg L^−1^) and temperature (27–47°C). Scanning electron microscopy (SEM) was used to examine external specifications of adsorbent morphology. An FTIR spectrophotometer was used to study the functional groups on the biaxial surface. The adsorption percentage was calculated by using the following equation [25]:

(1) $$\text{Removal}\;\left(\%\right)\;=\;\left(C_{0}-C_{e}\right)/\;\left(C_{0}\right)\;\times \;100$$

The amount of methyl orange in the adsorbent mass unit was determined using equation [26]:

(2) $$qe\;=\;\left(C_{0}-C_{e}\right)/M\times \;V$$

where *C*_o_ indicates the initial concentration of specified species (mg L^−1^), *C*_e_ indicates the equilibrium concentration of specified species (mg L^−1^), *V* indicates the solution's volume (L) and *M* indicates the used adsorbent's mass (g).

## 3 RESULT AND DISCUSSION

### 3.1 Characterisation of the adsorbent

**FIGURE 2:**
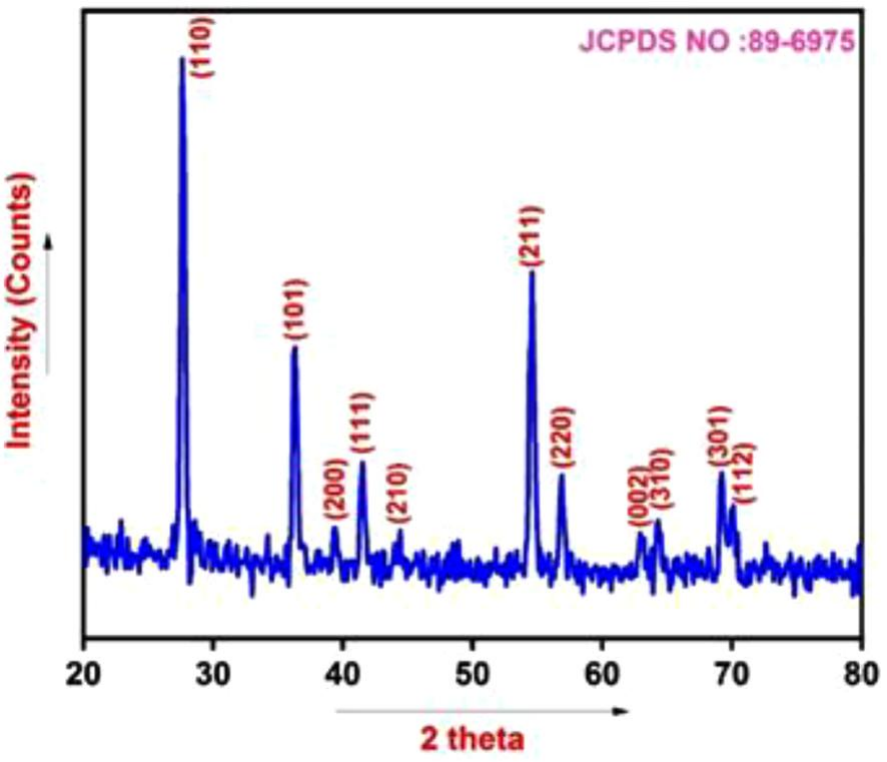
XRD pattern of green synthesis of TiO_2_ NPs

The images obtained using X‐ray diffractometer (BRUKER, Germany, Model‐D8‐Advance) along with Copper K radiation source ((*λ* = 1.54,060 A°) for prepared TiO_2_ NPs were illustrated in Figure 2 below. The characteristic XRD peaks of TiO_2_ crystalline reflections at 27.48°, 36.02°, 39.25°, 41.20°, 43.97°, 54.43°, 56.62°, 62.90°, 64.06°,68.96° and 69.50° corresponded to the (110), (101), (200), (111), (210), (211), (220), (002), (310), (301) and (112) planes, respectively. The X‐ray diffractometer peaks obtained were concurrent with JCPDS no. 89‐6975 (P42/nm space group) and thereby revealed a crystalline and tetragonal structure. The sharp peaks (1 1 0), (1 0 1) and (2 1 1) planes of anatase exhibited the synthesised TiO_2_ nanoparticles occurring in the anatase state and also revealed the highest purity of TiO_2_ nanocrystals thereby confirming the green synthesis of TiO_2_ nanoparticles in adsorbing the methyl orange dye effectively. In addition to the TiO_2_ NPs crystal structure, the below equation was also utilised to compute the crystallite sizes of TiO_2_ NPs [27].

(3) D=(0.9λ)/(βcosθ)

From the above Debye Scherrer's equation.

*D* = average particle size.

*λ* = wavelength of X‐ray,

*β* = Intensity of full width half maximum and.

*θ* = diffraction angle.

According to the Debye Scherrer's equation, the average crystallite size was noticed as 20.90 nm for the diffraction angle 36.02° with lattice parameters (*a* = 4.51 and *c* = 2.98).

**FIGURE 3:**
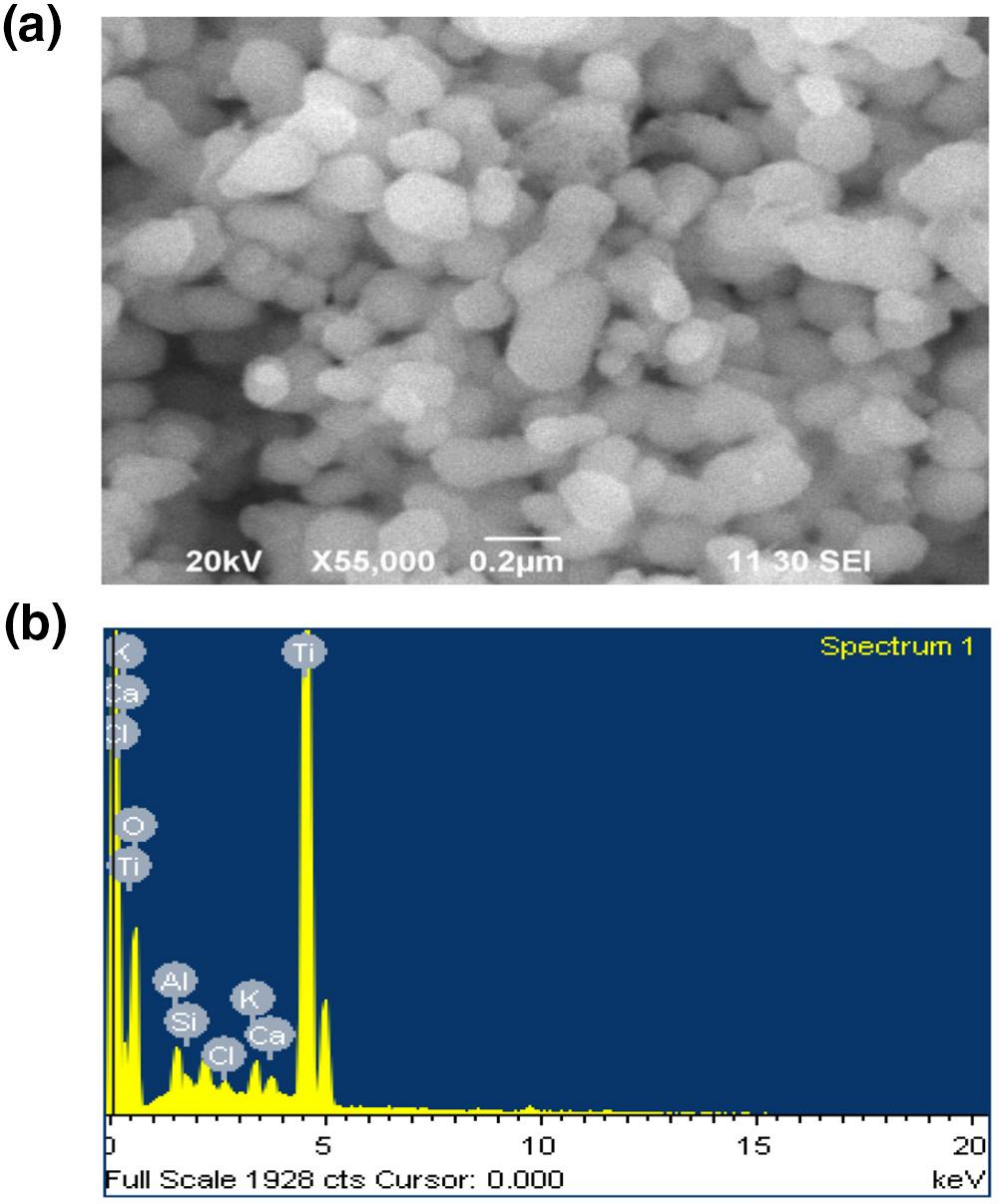
SEM and EDX image for green synthesis of TiO_2_ (a) SEM image of TiO_2_ and (b) EDAX image of TiO_2_

Figure 3a,b shows the SEM images and EDX spectra of the green synthesis of TiO_2_ NPs. The SEM image displayed an irregular, rough and heterogeneous porous structure responsible for the adsorption of the MO dye. The EDX spectra (Fig. 3b) confirmed the presence of aluminium, titanium, silicon; oxygen and calcium elements which were used for adsorption of MO dye. From these observations, it is revealed that there was a good agreement with the XRD results.

**FIGURE 4:**
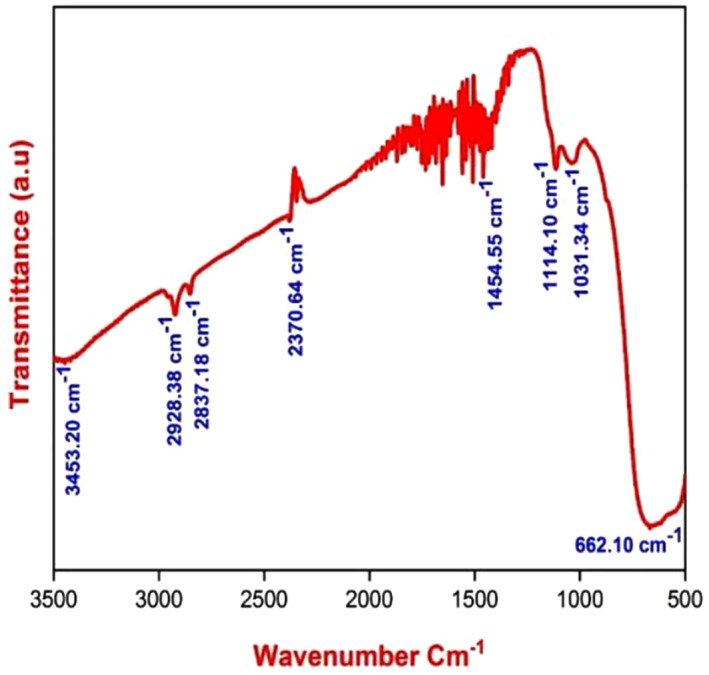
FTIR spectrum of TiO_2_ nanoparticles

The FT‐IR spectrum of green synthesis of TiO_2_ NPs is shown below in Figure 4 and revealed the following. In the wave numbers 3453.20 cm^−1^, 2928.38 cm^−1^, the stretching vibrations were noted and it was attributed to the presence of terminal alkynes and C–H stretch of aromatics and =C–H bond of alkenes and alkanes. The H–C=O and C–H stretching of aldehyde resulted in peaks at 2837.18, 2370.64 cm^−1^. The peaks at 1454.55 cm^−1^ confirmed the occurrence of aromatic ring in the sample. The peaks at 1114.10 cm^−1^ confirmed the occurrence of –CH_2_X of alkyl halides. The peak at 662.10 cm^−1^ confirmed the occurrence of the metal‐oxide (TiO_2_ in the composite) bond. The occurrence of hydroxyl and carboxylic acid groups were attributed for the adsorption process of MO dye onto the green synthesis of TiO_2_ NPs surface.

### 3.2 Effect of contact time

**FIGURE 5:**
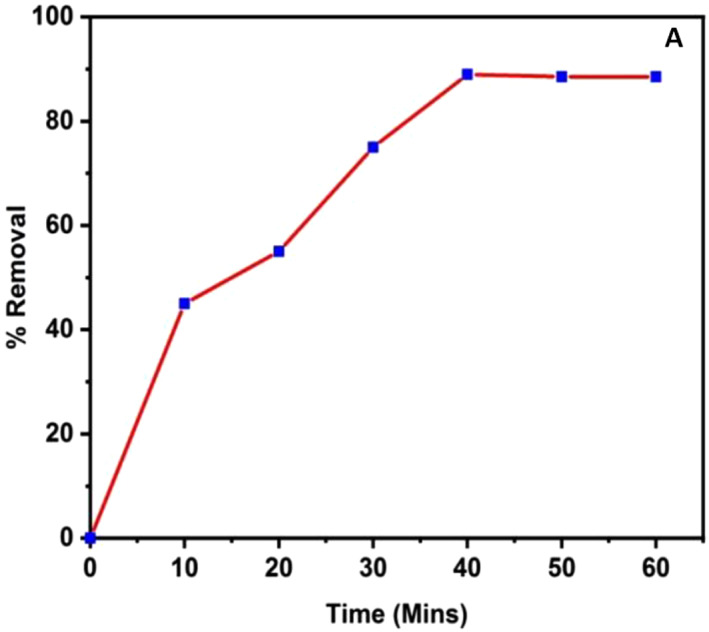
Effect of Contact time

To increase the efficiency of removal, methyl orange dye's rate of adsorption by the adsorbent was determined by reacting 10 mg L^−1^ of methyl orange dye solution in neutral pH with 0.01 g of TiO_2_ nanoparticles at different time intervals of 10 to 60 min, as illustrated in Figure 5.

As observed from Figure 5, the efficiency of MO dye adsorption was increased from 45% to 90 % as the contact time increased from 10 min to 40 min. No further increase in the MO dye adsorption efficiency was observed as the contact time was increased from 40 min to 60 min. Hence, for additional experiments, the contact time was chosen as 40 min.

### 3.3 Effect of pH

**FIGURE 6:**
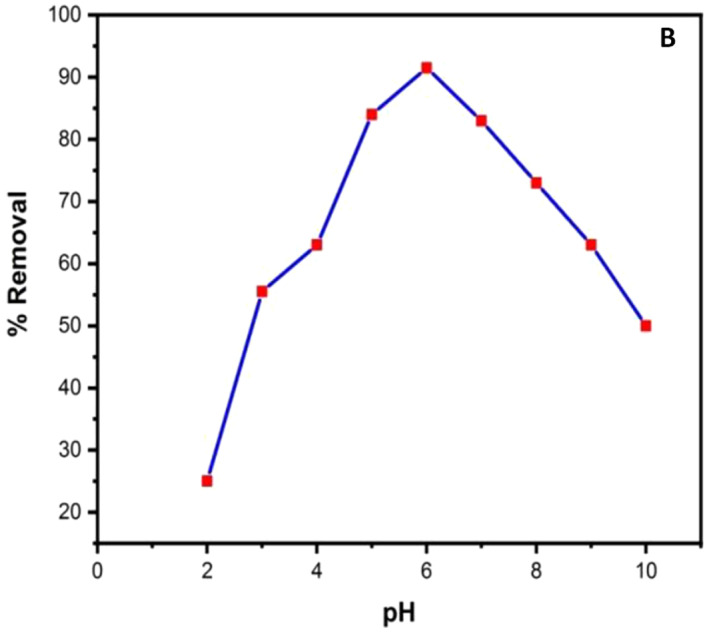
Effect of pH

The pH of the solution is also a significant parameter that influences adsorbents’ surface charge and degree of ionization of various contaminants and pollutants. Adsorptive process is affected by pH change evidenced by the dissociation of functional groups on the adsorbent active sites. [28]. By maintaining all the other parameters as constant, the influence of pH for the adsorption rate of MO by TiO_2_ NPs was observed at various pH ranging from 3.0 to 10.0 (Figure 6). Increased removal percentage of MO from 55.5 to 91.5 was observed with increased solution pH from 3.0 to 6.0 and later the adsorption efficiency was decreased. The electrostatic attraction between positively charged adsorbent surface and negatively charged dye molecules was attributed to the increased removal of MO with increment of pH from 3.0 to 6.0 [29]. The percentage removal of MO was decreased with increased pH beyond six which was attributed to electrostatic repulsion between negative‐charged adsorbent surface and positive‐charged dye molecules. Hence the optimum pH was considered as 6.0 for further procedures.

From the adsorption mechanism, initially methyl orange was liquefied after the SO^3−^ group from methyl orange that was separated and changed into anionic dye ions.

(4) $${R-\text{SO}}_{3}\;\text{Na}+H_{2}\;O\to {R-\text{SO}}^{3}+\;{\text{Na}}^{+}\;$$

Subsequently, the adsorption process is done owing to electrostatic attractions between the adsorbent outward and the anion of methyl orange.

(5) $${\text{RNH}}_{3^{+}}+\;{\text{RSO}}^{3-}\;\to \;{\text{RNH}}_{3^{+}}O_{3}\text{SR}\;$$

Conversely, the adsorption damaged with raising the value of methyl orange solution pH.

The lowering value of methyl orange adsorption can be clarified by the competition of the abundant presence of hydroxyl ions in basic solution for adsorption sites with MO anions. Meanwhile this adsorbent exhibited a strong adsorption at natural pH, the methyl orange pH values were unaltered for more investigates without exceptional commands.

### 3.4 Effect of adsorbent dose

**FIGURE 7:**
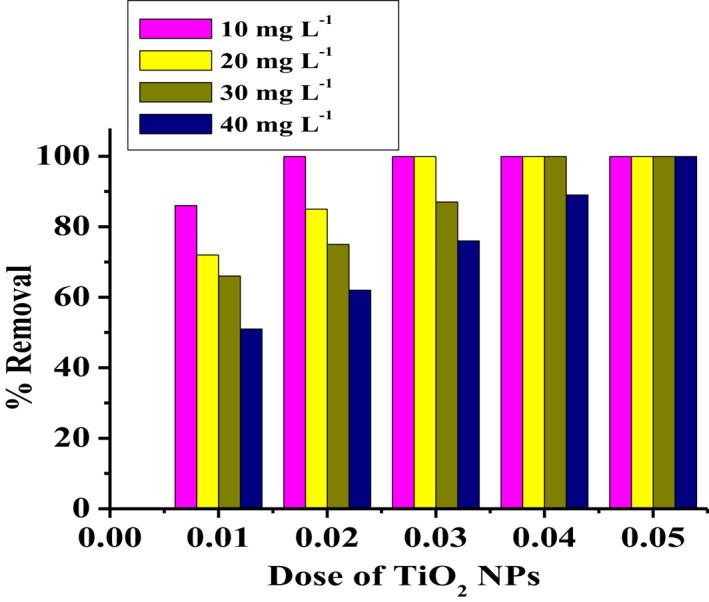
Effect of TiO_2_ NPs dose

To study the effect of adsorbent dose on the removal of MO dye, the experiment was carried out using 25 ml of MO solution containing an initial concentration of 10‐ 40 mg L^−1^ and the TiO_2_ NPs dose varied from 0.01‐0.04 g while maintaining optimum pH and contact time as 6.0 and 40 min, respectively (Figure 7). From the Figure 7 it was observed that the adsorption efficiency increased with increase in TiO_2_ dose and the maximum MO dye removal efficiency of 99.5 % was achieved at a dosage of 0.02 g in 10 mg L^−1^ MO concentration. The increased adsorption efficiencies with increasing adsorbent dose may be attributed to the higher surface area and the greater availability of active sites. Thus, a dose of 0.02 g was selected as the optimum adsorbent dose for MO dye adsorption.

### 3.5 Isotherms modelling

The characteristics of adsorption were verified utilising various adsorption isotherm models, to find the best fitted isotherm. In various concentrations (10‐60 mg L^−1^), the data derived were fitted to non‐linear isotherm models at 300, 310 and 320 K. The two and three parameters models (Freundlich, Langmuir, Redlich–Peterson and Dubinin–Radushkevich) were applied to evaluate the fit by isotherm for the adsorption of dyes. By using MATLAB R2010b, the isotherm parameters were determined. The Freundlich adsorption isotherm can be used to define heterogeneous systlows [30]:

(6) $$\;qe=K_{F}C_{e}^{1/n}$$

where *K*_F_—Freundlich constant ((mg g^−1^) (L mg^−1^)^(1/*n*)^) is related to the bonding energy. *n* (g L^−1^)—a deviation measure from linearity of adsorption. *n* value—the degree of non‐linearity between solution concentration and adsorption as follows: if *n* = 1, adsorption is linear; if *n* < 1, adsorption is finalised as a chemical process; if *n* > 1, adsorption is finalised as a physical process. The Langmuir model is implemented under the ideal assumption of the totally homogenous adsorption surface and represented as follows [31]:

(7) $$qe=\frac{q_{m}K_{L}C_{e}}{1+K_{L}C_{e}}$$

where *q*_m_ (mg g^−1^) is the maximum monolayer adsorption capacity, and *K*_L_ (L mg^−1^) Langmuir constant relating to adsorption energy. The essential characteristics of the Langmuir isotherm parameters can be used to predict the affinity between the sorbate and sorbent using the separation factor or dimensionless equilibrium parameter, ‘RL’, expressed as in the following equation [32]:

(8) $$\text{RL}=\frac{1}{1+K_{L}C_{0}}$$

The value of separation parameter RL provides important information about the nature of adsorption. The Redlich‐Peterson isotherm is an empirical isotherm incorporating three parameters. It combines both the Langmuir and Freundlich equations. while the mechanism of adsorption is a hybrid and does not follow ideal monolayer adsorption. The equation is given as [33]:

(9) $$qe=\frac{K_{R}C_{e}}{1+a_{R}C_{e}^{g}}$$

where *K*_R_ (L g^−1^) and *a*_R_ (L g mg‐g) are Redlich‐Peterson isotherm constants and *g* is an exponent that lies between 0 and 1. For *g* = 1, the equation converts to the Langmuir isotherm; for *g* = 0, it simplifies to Henry's law equation; and for1<< *a*_R_ $C_{e}^{g}$ it is identical with the Freundlich isotherm. The Dubinin‐Radushkevich isotherm is applied to find out the adsorption mechanism based on the potential theory assuming a heterogeneous surface and is expressed as follows [34]:

(10) $$qe=q_{m}De^{-\beta \varepsilon 2}$$

where *q*_m_ *D* (mg/g) is the Dubinin‐Radushkevich monolayer capacity, *β* is a constant related to sorption energy, and *ε* is the Polanyi potential which is related to the equilibrium concentration as follows [35],

(11) $$\varepsilon =RT\;\ln\left[1+\frac{1}{C_{e}}\right]$$

where *R* is the gas constant (8.314 J/mol K) and *T* is the absolute temperature. The constant *β* gives the mean free energy, *E*, of sorption per molecule of the sorbate when it is transferred to the surface of the solid from infinity in the solution and can be computed using the relationship [36]:

(12) $$E=\left[\frac{1}{\sqrt{2\beta }}\right]\;$$

Adsorption mechanism type was determined by the magnitude of *E*. Upon transferring 1 mol of ion to the adsorbent surface, if the value of *E* is less than 8 kJ/mol then it indicated physical adsorption, if the value of *E* is between 8 and 16 kJ/mol then it indicated the ion‐exchange adsorption [37], whereas if the value of *E* exists in the range of 20‐40 kJ/mol then it indicated chemisorption [38].

**FIGURE 8:**
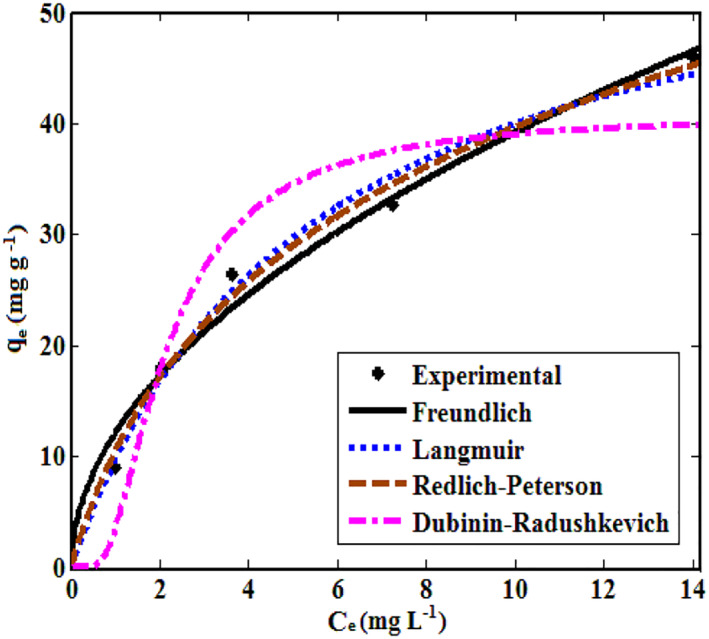
Non‐linear isotherm plots for adsorption of MO onto TiO_2_ NPs

The isotherm constants, correlation coefficients (*R* ^2^), sum of squares error (SSE) and root‐mean‐squared error (RMSE) values were estimated from the plot of *q*_e_ versus *C*_e_ (Figure 8) and are listed in Table 1. The *R* ^2^ values were closer to 1 and small SSE, RMSE values indicated better curve fitting. A better fit for TiO_2_ NPs was exhibited by the Langmuir isotherm model based on the *R* ^2^, SSE and RMSE values from Table 1. Hence, the monolayer adsorption of methyl orange was confirmed by the good fit of equilibrium data for TiO_2_ NPs in Langmuir isotherm expression.

**TABLE 1:** Isotherm parameters for the absorption of MO onto TiO_2_ NPs

Isotherm model	Parameter	TiO_2_ NPs
Freundlich	*K*_F_(mg/g)	9.633
	N	2.631
	*R* ^2^	0.943
	SSE	24.11
	RMSE	2.455
Langmuir	*q*_m_(mg/g)	273.37
	K_L_ (L/mg)	0.188
	*R* ^2^	0.990
	SSE	3.08
	RMSE	0.857
	RL	0.347‐0.081
Redlich‐	*K*_R_(L/g)	8.793
Peterson	*a*_R_(L/mg)	0.319
	*G*	0.907
	*R*_2_	0.969
	SSE	13.79
	RMSE	2.088
Dubinin‐	*q*_mD_(mg/g)	28.32
Radushkevich	*β*(mg^2^/J)	1.854 × 10^−7^
	*E* (kJ/mol)	1.642
	*R* ^2^	0.773
	SSE	95.52
	RMSE	4.887

Furthermore, a favourable adsorption of MO on TiO_2_ NPs was noticed since the RL values for the Langmuir isotherm fall between 0 and 1 (Table 1).

### 3.6 Adsorption kinetics of dyes onto TiO_2_ NPs

The kinetic studies were done to investigate the mechanism of adsorption at various stages of migration of molecules of the adsorbate from the bulk solutions onto the adsorbent surface. The pseudo‐first‐order and pseudo‐second‐order models were used to observe the kinetics of methyl orange adsorption onto TiO_2_ NPs thereby investigating the controlling mechanism like chemical reaction and mass transfer.

A pseudo‐first‐order equation of Lagergren [39] is generally expressed as:

(13) $$\log\left(q_{e}-q_{t}\right)=\log\;q_{e}-\frac{k_{1}}{2.303}\;t$$

where *q*_e_ (mg g^−1^), *q*_t_ (mg g^−1^) are the adsorption amount at equilibrium and at time *t* (min), respectively. *k*_1_ (min^−1^) is the rate constant of the pseudo‐first‐order adsorption process. The constants were determined experimentally by plotting of log(*q*_e_ − *q*_t_) versus *t*. Pseudo‐second‐order model was also generally applied to fit the experimental data. The linear form of pseudo‐second‐order model [40] can be expressed as:

(14) $$\frac{t}{q_{t}}=\frac{1}{k_{2}q_{e}^{2}}+\frac{t}{q_{e}}$$

where *k*_2_(g mg^−1^min^−1^) is the rate constant of adsorption. By plotting a curve of *t*/*q*_t_ against *t*, *q*_e_ and *k*_2_ can be evaluated. Where *q*_e(exp)_ and *q*_e(cal)_ are experimental and calculated value of methyl orange adsorbed on the adsorbents, SSE is the sum of square error. It was confirmed that a low value of sum of square error was better than the fit. All the kinetic parameters, correlation coefficients and SSE are listed in Table 2.

**TABLE 2:** Kinetic parameters for removal of MO onto TiO_2_

Kinetic model	Parameters	Concentration (mg L^−1^)		
		10	20	30
Pseudo‐First‐order	*q* ^exp^ (mg g^−1^)	9.5	18.0	24.60
	*k*_1_ (min^−1^)	0.0187	0.0238	0.0327
	*q* ^cal^ (mg g^−1^)	6.17	12.70	14.50
	*R* ^2^	0.978	0.967	0.954
	SSE	9.95	17.54	27.4
Pseudo‐second o‐rder	*k*_2_ (g mg^−1^ min^−1^)	0.030	0.028	0.043
	*q* ^cal^ (mg g^−1^)	9.4	17.6	24.30
	*R* ^2^	0.998	0.997	0.994
	SSE	0.052	0.087	0.076
Intra‐particle diffusion	*K*_d_	0.658	1.55	1.77
	(mg g^−1^min^−1/2^)			
	*C* (mg g^−1^)	9.1	16.7	22.20
	*R* ^2^	0.973	0.951	0.943

For the pseudo‐second‐order model, the correlation coefficient (*R* ^2^) was much nearer to unity. The calculated *q*_e_ value and the experimental *q*_e_ value were observed to be closer. Furthermore, in the event of pseudo‐second order, the SSE value was also observed to have a lower value. These outcomes confirmed that the pseudo‐second‐order equation has mainly governed the adsorption kinetics of MO onto the TiO_2_ NPs. This confirmed that the chemical process involving the sharing of electrons or covalent forces through exchange of electrons between adsorbent and adsorbate controlled the overall rate of methyl orange adsorption process. To understand the detail mechanism of adsorption, the kinetic data was further analysed by the following intra‐particle diffusion model equation [41]:

(15) $$q_{t}\;=\;k_{d}\;t1/2\;+\;C$$

where *q*_t_ is the amount of adsorbate adsorbed at time t (min) in mg g^−1^, *k*_d_ is the intra‐particle diffusion constant (mg L^−1^min^−1^), *C* mg g^−1^ is the intercepts which provide information about the thickness of the boundary layer. The value of *k*_d_ and *C* were evaluated from the slope and intercepts plot of *q*_t_ versus *t*1/2 and shown in Table 2.

In the event of intra‐particle diffusion, if the graph of *q*_t_ versus *t*1/2 was linear and passed through origin then the rate‐limiting step would be intra‐particle diffusion, otherwise, film diffusion would be controlling the sorption technique. The multi‐linearity plot obtained by plotting the intra‐particle mass transfer diffusion indicated that the adsorption process of MO on TiO_2_ NPs was very much influenced by two portions. Among the two portions, the initial portion was allotted to the bulk diffusion or film diffusion and the second portion was allotted to the intra‐particle diffusion where the intra‐particle diffusion was not only the sole rate‐controlling step for adsorption but also was dominated by both the pore and film diffusion process.

### 3.7 Thermodynamics

To study the possibility of adsorption techniques and thermodynamic parameters, the removal efficiency of MO on TiO_2_ was investigated at various temperatures of 303, 313  and 323 K. Thermodynamic parameters such as Gibb's free energy (∆*G*°), enthalpy (∆*H*°) and entropy (∆*S*°) can be computed using the below equation [42].

(16) $$\Delta G^{0}=\;\Delta H^{0}-\;T\Delta S^{0}$$

(17) $$\ln\;Kc=\frac{\Delta S^{0}}{R}\;-\;\frac{\Delta H^{0}}{RT}$$

In Table 3, the thermodynamic parameters values are presented. As per the experiment, the adsorption process was confirmed as endothermic by the positive value of ∆H° and was also confirmed as spontaneous and feasible by the negative value of ∆G°.

**TABLE 3:** Thermodynamic parameters for adsorption of MO on TiO_2_ NPs

Temperature (K)	∆*G*°	∆*H*°	∆*S*°
	(kJ mol^−1^)	(kJ mol^−1^)	(kJ mol^−1^K)
303	−4.72		
313	−5.89	55.05	0.376
323	−7.25		

During adsorption, good affinity of TiO_2_ NPs for MO and greater randomness in the solid‐liquid interface was confirmed by the positive value of ∆S°. The combination of two processes were involved in the adsorption of solid‐liquid system, the first process included the adsorption of the dye molecule on the adsorbent surface ensuring the removal and dispersion of previously adsorbed water molecule bonded with the dye into the solution thereby increasing the entropy and the second process involved the adsorbent of the adsorbate molecule. Increased degree of freedom of adsorbed species was indicated by the positive value of ∆S°_._

### 3.8 Desorption and regeneration studies

In the industrial waste water treatment, adsorbent's regeneration is vital and its desorption rate confirms the effective adsorbent's reuse. To calculate the effective regeneration and reuse of TiO_2_ NPs, experiments on desorption were performed in a batch mode utilising four various eluents like 0.001, 0.01, 0.1, 0.5 and 1 M nitric acid. It was confirmed that efficiency of desorption utilising 0.001, 0.01, 0.1, 0.5 and 1 M were 75.78%, 86.50%, 98.78%, 99.57% and 99.78% respectively. On utilising different adsorption‐desorption processes, the adaptive reuse of TiO_2_ NPs was obtained for five eluents and the optimal desorption rate was confirmed at 0.1 M, further that no noteworthy desorption rate was noticed. Hence 0.1 M of HNO_3_ can be used for efficient regeneration of dye saturated TiO_2_ NPs for the subsequent adsorption experiments.

## 4 CONCLUSION

In this research, green synthesis of TiO_2_ NPs from *Phyllanthus niruri* leaf extract by sol‐gel method showed 99.5% removal of MO from aqueous solution by the adsorption process and is observed at a lesser concentration of 10 mg L^−1^. The pH of the solution decided the adsorption rate and higher removal efficiency was observed at a pH of 7.0 and the removal efficiency was managed by the pseudo‐second‐order kinetic model. The data of 5y3 nadsorption isotherms confirmed that the Langmuir isotherms model suited well for the adsorption technique. At 303 K, the adsorption volume of TiO_2_ NPs was noted as 273.37 mg g^−1^. The tehermodynamic studies confirmed that the adsorption technique was spontaneous, feasible, and endothermic in nature. The optimal eluent for adsorbent recycling process was confirmed as 0.1 M HNO_3_. The results obtained confirmed that green synthesis of TiO_2_ NPs synthesised from *Phyllanthus niruri* leaf extract was a nominal and best adsorbent for MO removal from aqueous solution.
